# Carbonic anhydrase 9 is a predictive marker of survival benefit from lower dose of bevacizumab in patients with previously treated metastatic colorectal cancer

**DOI:** 10.1186/1471-2407-9-246

**Published:** 2009-07-21

**Authors:** Yong Sang Hong, Hyeon Jin Cho, Sun Young Kim, Kyung Hae Jung, Ji Won Park, Hyo Seong Choi, Jae Hwan Oh, Byung Chang Kim, Dae Kyung Sohn, Dae Yong Kim, Hee Jin Chang

**Affiliations:** 1 Center for Colorectal Cancer, Research Institute and Hospital, National Cancer Center, Goyang, Republic of Korea; 2 Division of Hematology and Medical Oncology, Department of Internal Medicine, Seoul National University Hospital, Seoul National University College of Medicine, Seoul, Republic of Korea

## Abstract

**Background:**

Carbonic anhydrase 9 (CA9) is a marker for hypoxia and acidosis, which is linked to a poor prognosis in human tumors. The purpose of this comparative analysis was to evaluate whether CA9 and VEGF expression are associated with survival outcomes in patients with metastatic colorectal cancer (mCRC) after treatment with bevacizumab as second or later line treatment.

**Methods:**

Thirty-one mCRC patients who were treated with bevacizumab-containing chemotherapy as second or later line treatment and who had analyzable tumor paraffin blocks were selected for this study. The planned dose of bevacizumab was 5 mg/kg/2-week. Immunohistochemical (IHC) staining of CA9 and VEGF was performed and their expression was scored by the intensity multiplied by percentage of stained area.

**Results:**

The overall response rate was 19.4% and the disease control rate (DCR) was 61.3% with 6 partial responses and 13 cases of stable disease. The DCR was significantly higher in patients with a lower CA9 expression score compared to those with a higher score (80.0% vs. 27.3%, respectively, P = 0.004). The patients with a low CA9 expression score also showed better outcomes with regard to the median progression-free survival (P = 0.028) and overall survival (P = 0.026). However, VEGF expression was not associated with the DCR and survival.

**Conclusion:**

Lower degree of CA9 expression was associated with better clinical outcomes in patients with mCRC treated with lower dose bevacizumab-based chemotherapy. Prospective studies are now needed to determine the correlation between CA9 expression and clinical outcomes after bevacizumab treatment, at different doses and in varied settings.

## Background

For the patients with metastatic colorectal cancer (mCRC), 5-fluorouracil (5-FU) based chemotherapy has been the standard regimen [1,2]. Since the late 1990's, combination chemotherapy with 5-FU/leucovorin (LV) plus oxaliplatin (FOLFOX) or irinotecan (FOLFIRI) has been shown to improve the response rates and survival when used as either first-line or second-line treatment [3-5]. These combination regimens had been the treatment of choice for patients with mCRC before the introduction of bevacizumab.

Bevacizumab, a recombinant humanized monoclonal antibody, targets vascular endothelial growth factor (VEGF), and prevents its interaction with receptors on the vascular endothelial cells that mediate angiogenesis; a process critical for tumor progression [6,7]. Since the successful results of a landmark study were published in 2004 [8], bevacizumab has been proven to be effective in several clinical trials when combined with various cytotoxic chemotherapeutic agents in patients with metastatic disease as first-line or neoadjuvant treatment before metastasectomy [9-14]. Thus, bevacizumab plus 5-FU based regimens are highly recommended in previously untreated patients with mCRC. Bevacizumab-containing combination chemotherapy also was proved to be effective as second-line treatment in a phase III trial, E3200 study; however, the approved dose for previously treated patients based on the results from E3200 trial is twice as high as that of first line treatment [15].

Tumor hypoxia is known to be associated with treatment failure in several malignancies. Carbonic anhydrase 9 (CA9) is one of the representative markers for tumor hypoxia; it is a transmembrane protein that plays a major role in the adaptation and proliferation of cells, in hypoxic and acidic conditions, by regulating the intracellular and extracellular pH [16,17]. CA9 was initially identified in HeLa cells [18]; its expression has been found in a variety of tumor types including colorectal cancer [19]. Hypoxia is one of driving forces of tumor angiogenesis; therefore, expression of the hypoxia-inducible enzyme, CA9, might be associated with the outcome of antiangiogenic treatment.

In this study, we aimed to investigate the efficacy of lower dose bevacizumab (5 mg/kg/2-wk), a half of approved dose for second-line setting, for pretreated patients. In addition, clinicopathologic analysis was done to evaluate the correlation between expression of CA9/VEGF and efficacy of bevacizumab-containing regimen.

## Methods

### Patients and tissue samples

From July 2005 to October 2008, 50 patients with previously treated mCRC who received a lower dose of bevacizumab were identified from a prospective medical oncology patient database at the Center for Colorectal Cancer, National Cancer Center, Korea. Among them, study patients were selected according to the following inclusion criteria: 1) patients that were exposed and refractory to previous chemotherapy for metastatic disease prior to treatment with bevacizumab; 2) one or more unidimensionally measurable lesion(s) according to the RECIST (Response Evaluation Criteria in Solid Tumors) criteria [20] should be present; 3) planned dose of bevacizumab should not be in excess of 5 mg/kg/2-wk; and 4) adequate tumor paraffin blocks for immunohistochemical (IHC) staining should be available. Thirty one patients were finally included in this study.

This study was conducted in accordance with the Helsinki declaration and patients were provided with informed consent prior to receiving the study treatment. Additional informed consents for IHC staining were also obtained where appropriate. The protocol was approved by the Institutional Review Board of the National Cancer Center, Korea (protocol number NCCNCS-08-120).

IHC staining for CA9 and VEGF expression was evaluated by one pathologist (H. J. Chang) without knowledge of the clinical findings. The comparative analysis of the clinical results and CA9 and VEGF expression profiles was performed by medical oncologists (Y.S. Hong and H.J. Cho).

### Study treatment

Bevacizumab was administered at a dose of 5 mg/kg every 2 weeks or 7.5 mg/kg every 3 weeks on day 1, according to the schedule of the concomitant chemotherapy regimen. Concomitant cytotoxic chemotherapy regimens were chosen by the attending physicians and included the followings: 2-week scheduled concomitant cytotoxic chemotherapy with LV/5-FU (LV 200 mg/m^2 ^d1, 5-FU 400 mg/m^2 ^d1 and 5-FU 1200 mg/m^2^/day continuous infusion d1–2), FOLFIRI (irinotecan 180 mg/m^2 ^d1 and LV/5-FU as above) and FOLFOX (oxaliplatin 85 mg/m^2 ^d1 and LV/5-FU as above), and a 3-week scheduled concomitant regimen with oral fluoropyrimidines alone (capecitabine 2500 mg/m^2^/day or S-1 70 mg/m^2^/day d1–14). Dose modifications of bevacizumab were not considered, but delays of bevacizumab administration were permitted in synchrony with the schedule of other cytotoxic chemotherapy. Dose modifications of combined cytotoxic drugs were made for hematological or non-hematological toxicity on the basis of the most severe grade of toxicity that occurred during the previous cycle. Patients were treated until disease progression, development of unacceptable toxicity or patient refusal.

### Assessments of efficacy and toxicity

Objective tumor responses were assessed every 6 weeks using RECIST criteria; all responses required confirmation at 4 weeks or later. Toxicities were graded according to the National Cancer Institute Common Toxicity Criteria, version 3.0 (NCI-CTC 3.0).

### IHC stain for CA9 and VEGF

**Figure 1:**
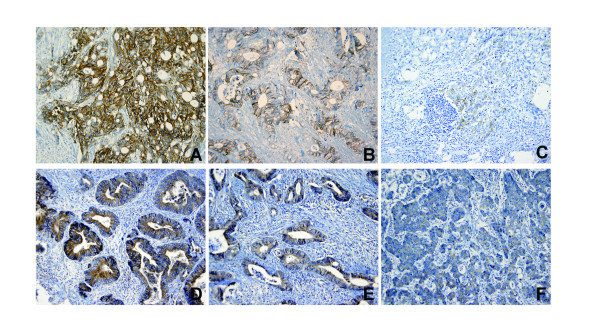
**Immunohistochemical expression of CA9 (A to C) and VEGF (D to F) of strong (A and D), moderate (B and E), and weak (C and F) intensities**.

**Figure 2:**
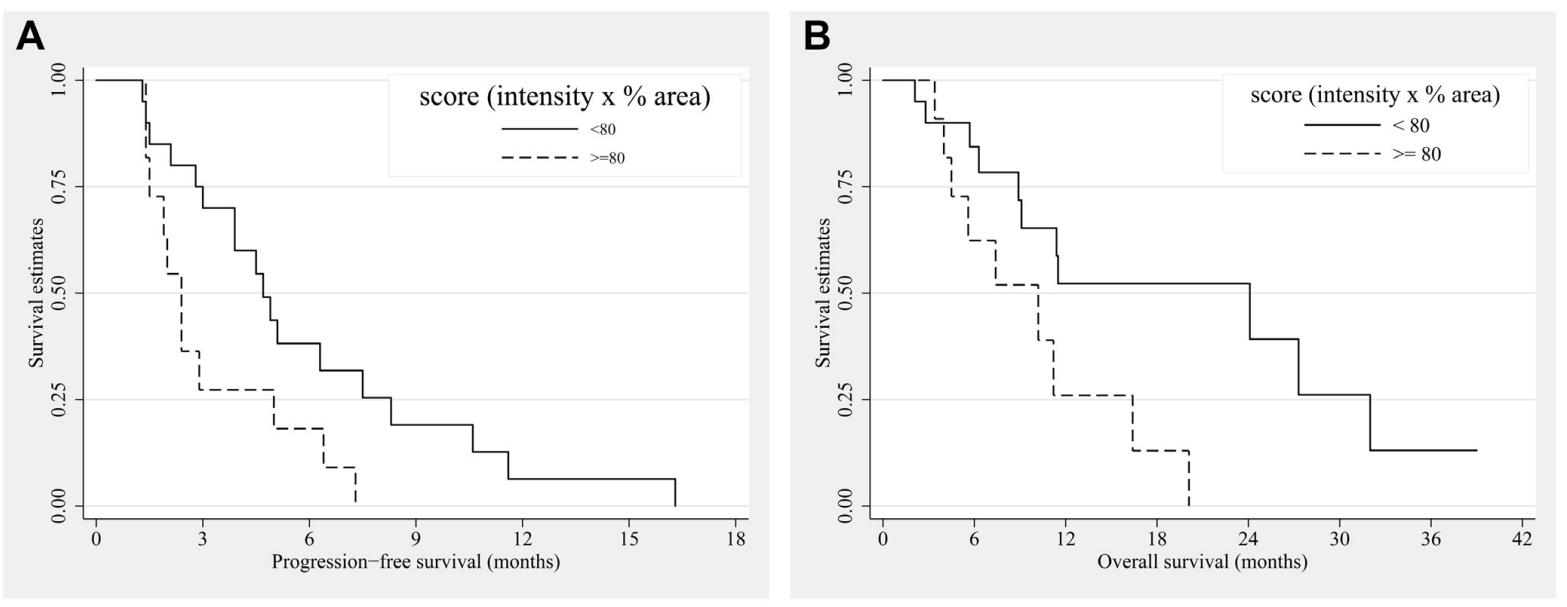
**Survival outcomes according to the CA9 expression scores**. The median PFS was 3.9 months and median OS with bevacizumab was 11.4 months; after the 17.6 months of median follow up of all 31 patients. There were statistically significant differences between the 2 groups according to the score in terms of median PFS (4.7 mo *vs. *2.4 mo, *p *= 0.028) and median OS (24.1 mo *vs. *10.2 mo, *p *= 0.026).

Immunostaining was performed using the labeled streptavidin-biotin complex (LSAB) method, with primary antibodies to CA9 (NB100-417, Novus Biologicals, CO, USA: dilution 1:1000) and VEGF (G153-694, BD Pharmingen, CA, USA: dilution 1:500). Formalin-fixed, paraffin-embedded sections (4-μm thick) were dewaxed for 15 minutes in xylene and hydrated by passage through a graded ethanol series to tap water. Antigen retrieval was performed by incubation in a citrate buffer solution (Antigen Unmasking Solution, Vector Laboratories, Burlingame, CA., USA) for 15 minutes in an 800-W microwave oven. Reaction products were not detected when non-immune serum or phosphate-buffered saline (PBS) was used instead of the primary antibodies. Positive expression was classified as unequivocal brown staining of the cell membranes (for CA9) or cytoplasm (for VEGF) of the tumor cells (Figure 1). Immunohistochemical expression was recorded as follows: 1) the intensity of the stain defined as 0 for negative, 1+ for weak, 2+ for moderate, and 3+ for strong (Figure 1); 2) the percent area was defined as the percentage of stained tumor cells in the entire tumor field; 3) the expression score was defined as the intensity multiplied by the percent area positive for tumor. A high CA9 and VEGF expression was defined by an expression score ≥ 80.

### Statistical analysis

All patients that showed adequate CA9 and VEGF IHC results and received at least one course of bevacizumab therapy were included in the survival analysis. Descriptive statistics were reported as proportions and medians. The chi-square test and the Fisher's exact test were used to compare proportions. Usually a chi-square test was used, but a Fisher's exact test was used if the expected value of any of the cells of the contingency table was below 5. The Wilcoxon rank-sum test was used to compare medians between the patient groups. The overall survival (OS) and the progression free survival (PFS) were assessed by the Kaplan-Meier method and the 95% confidence interval (95% CI) for the median time to an event was computed. The log-rank test was used to compare survival outcomes of the patient groups and the Cox proportional hazards model was also used to examine the factors associated with survival. The OS was measured from the start of bevacizumab therapy or from the date of first line chemotherapy until death, censoring patients that had not died at the date of the last follow up. The PFS was defined as the time from the date of bevacizumab treatment to the date of disease progression or death by any cause, censoring patients without progression at the date of the last disease assessment. Analyses were performed using Stata version 10.0 (Stata Corp, College Station, Texas, USA).

## Results

### Patient characteristics

Between July 2005 and October 2008, 31 patients that received bevacizumab as second or later line treatment, and that had tumor paraffin blocks available for immunostaining were selected for the study; the baseline patient characteristics are described in Table 1. The median age was 51.6 years (range: 31.8–67.8), 15 patients (48.4%) were male and 19 patients (61.3%) had colon cancer as their primary malignancy. Twenty-eight patients (90.3%) had a good performance status (ECOG 0 or 1). The most frequent site of metastasis was to the liver (17/31, 54.8%). All 31 patients were exposed to three effective cytotoxic drugs (fluoropyrimidines, irinotecan and oxaliplatin) during their entire treatment period and 21 patients (67.7%) were also exposed to cetuximab.

**Table 1:** Patient characteristics and treatment responses

		Score of CA 9 expression		
			
Patient characteristics (n = 31)		<80	≥ 80	P value
		(n = 20)	(n = 11)	
Age, median (range)	51.6 (31.8 – 67.8)	52.6 (33.3 – 66.7)	50.4 (31.8 – 67.8)	0.741*

Gender				
Male	15	10	5	0.809^†^
Female	16	10	6	

Performance status				
0	3	2	1	1.000^‡^
1	25	16	9	
2	3	2	1	

Line of bevacizumab				
2^nd ^line	13	9	4	0.718^‡^
≥ 3^rd ^line	18	11	7	

Primary sites				
Rectum	12	9	3	0.452^‡^
Colon	19	11	8	

Site of distant metastasis				
Liver	17	10	7	0.465^†^
Lymph node	15	12	3	0.081^†^
Lung	14	9	5	0.981^†^
Omentum	11	9	2	0.241^‡^
Bone	7	6	1	0.372^‡^

Sum of metastatic sites	4	3	1	
< 3	20	13	7	1.000^‡^
≥ 3	11	7	4	

Combined chemotherapy				
FOLFOX	15	10	5	0.439^‡^
FOLFIRI	6	5	1	
Fluoropyrimidines alone	10	5	5	

Treatment responses				
Complete response	-	-	-	
Partial response	6 (19.4%)	4	2	
Stable disease	13 (41.9%)	12	1	
Disease control rate^§^	19 (61.3%)	16 (80.0%)	3 (27.3%)	0.004^‡^
Progressive disease	11 (35.5%)	3	8	
Not evaluable	1 (3.2%)	1	0	

*P value for age was calculated with the Wilcoxon rank-sum test.

^†^P values for gender, liver metastases, lymph node metastases, and lung metastases were calculated with use of the chi-square test.

^‡^P values for performance status, line of bevacizumab, omental metastases, bone metastases, sum of metastatic sites, combined chemotherapy, and disease control rate were calculated with the Fisher's exact test.

^§^Disease control rate = complete response + partial response + stable disease

The CA9 expression was negative in 6 patients (19.4%), weakly positive in 1 (3.2%), moderately positive in 11 (35.5%), and strongly positive in 13 patients (41.9%). The mean value for the percent of stained area for the CA9 IHC was 30.1% (range, 0 – 95%) and the mean of expression score was 82.8 (range, 0 – 285). The patient characteristics according to the CA9 expression are shown in Table 1. There was no statistically significant differences in patient characteristics between the groups according to the CA9 expression scores, except for lymph node metastases, which tended to be less frequent in patients with higher CA9 expression (*P *= 0.081, by chi-square test). The intensity of VEGF expression was negative in 7 (22.6%), weakly positive in 9 (29.0%), moderately positive in 13 (41.9%), and strongly positive in 2 patients (6.5%). The mean percent of stained area for VEGF was 44.5% (range, 0 – 90%) and the mean of expression score was 83.1 (range, 0 – 270). Patient characteristics were also evenly distributed according to VEGF expression, except for more frequent omental metastases in patients with lower VEGF expression (data not shown).

### Treatment delivery, toxicity and responses

In total, 200 cycles of lower dose bevacizumab were administered with a median of 4.5 cycles per patient (range 1 – 16 cycles). Fifteen patients (48.4%) received FOLFOX as concomitant cytotoxic chemotherapy, 6 patients (19.3%) received FOLFIRI and 10 patients (32.3%) received fluoropyrimidines alone (5-FU/LV in 7, S-1 in 2 and capecitabine in 1 patient, respectively). These study treatments were the second line treatment in 13 patients (41.9%) and third or later line treatment in 18 patients (58.1%). Combined chemotherapy and the line of bevacizumab were balanced between groups with higher and lower CA9 expression scores, without significant differences. The median delivered dose intensity of bevacizumab was 4.2 mg/kg/2-wk (84.0%). The grade 3 bevacizumab-related toxicities per patient included: thromboembolism (1, 3.2%), bleeding (1, 3.2%) and hypertension (1, 3.2%); grade 2 included proteinuria (5, 16.1%), bleeding (1, 3.2%) and hypertension (2, 6.4%).

The overall response rate was 19.4% (95% CI, 5.5 – 33.3) and the disease control rate was 61.3% (95% CI, 44.2 – 78.4) with 0 complete response, 6 partial responses and 13 stable diseases (Table 1). There were statistically significant differences in the disease control rates; 80.0% (16/20) in patients with a CA9 expression score < 80 and 27.3% (3/11) in those with a CA9 expression score ≥ 80 (*P *= 0.004). By contrast, the disease control rate was similar in patients with lower VEGF expression compared to those with higher VEGF expression (60.0% vs. 62.5%, respectively. *P *= 0.919).

### Survival

**Figure 3:**
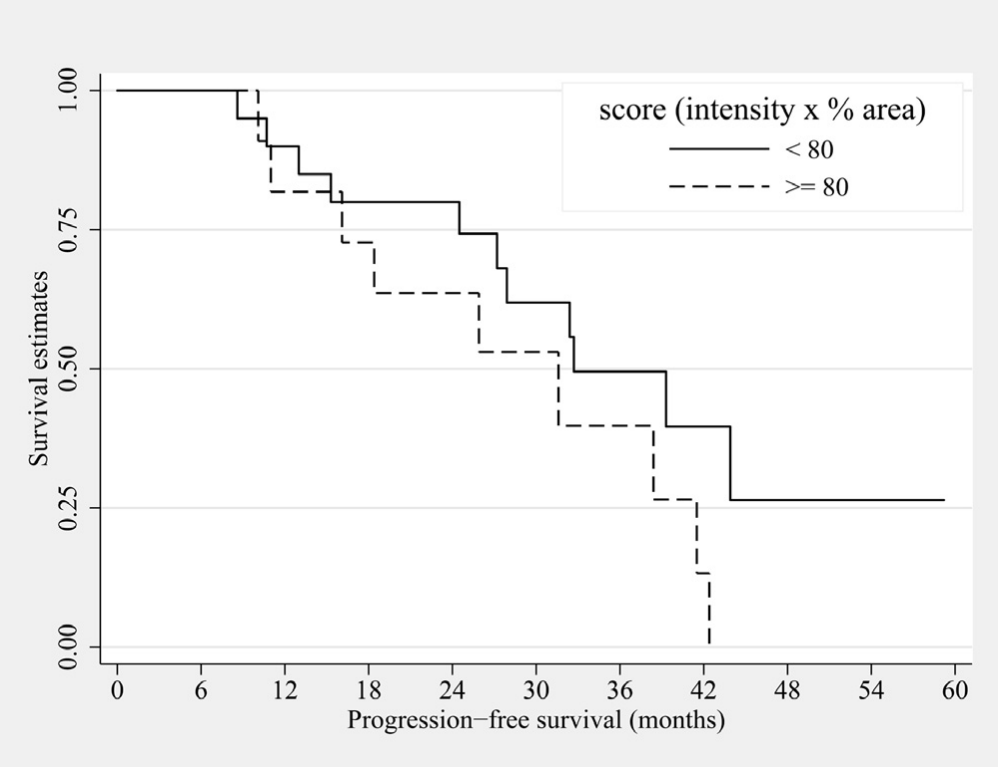
**The OS from initiation of first – line chemotherapy showed no statistical difference based on CA9 expression scores**.

The survival outcomes are shown in Figures 2 and 3. The median PFS was 3.9 months (95% CI, 1.7 – 6.1) and the median OS with bevacizumab was 11.4 months (95% CI, 9.5 – 13.4), after the median 17.6 months of follow up (range, 3.8 – 43.9). The median OS from initiation of first-line treatment was 32.4 months (95% CI, 25.7 – 39.1). The PFS was better, with borderline significance, in patients that received second line bevacizumab compared to those that received third or later line bevacizumab (5.0 vs. 2.4 months, hazard ratio (HR) 0.49 [95% CI, 0.23–1.05], *P *= 0.060); the OS was not statistically different according to the line of bevacizumab treatment (16.4 vs. 11.2 months, HR 0.67 [95% CI, 0.24–1.83], *P *= 0.426).

In a comparative analysis based on the CA9 expression scores, patients with a lower score (< 80) had improved PFS with bevacizumab when compared to those with a higher score (≥ 80) (4.7 months vs. 2.4 months, respectively; HR 0.42 [95% CI, 0.18–0.94], *P *= 0.028). A lower CA9 expression score was also associated with a better overall survival compared to higher scores; 24.1 months vs. 10.2 months, respectively (HR 0.35 [95% CI, 0.13–0.92], *P *= 0.026). However, the PFS and the OS did not differ between the subgroups based on higher or lower VEGF expression scores: the median PFS of patients with VEGF expression scores < 80 was 3.9 months, and those with higher VEGF expression scores had also 3.9 months of PFS (HR 1.54 [95% CI, 0.72–3.30], *P *= 0.25). The median OS was 9.1 months for patients with VEGF expression scores < 80, whereas it was 11.5 months for those with scores ≥ 80 (HR 1.66 [95% CI, 0.48–5.77], *P *= 0.68). The median OS from initiation of first-line treatment showed no statistically significant differences based on the CA9 expression scores (*P *= 0.164) or VEGF expression scores (*P *= 0.62).

The univariate analysis showed that there were five factors associated with the PFS with a *P *value < 0.1: the CA9 expression score, gender, the line of treatment with bevacizumab, presence of bony metastases, and number of metastatic sites. Multivariable analysis with the Cox regression hazard model revealed that the CA9 expression score and the number of metastatic sites were significantly associated with the PFS: the risk of progression was significantly increased among patients with higher CA9 scores compared to those with lower CA9 scores (HR = 3.50 [95% CI, 1.39–9.09]; *P *= 0.007) and for patients with 3 or more metastatic sites compared to those with 1 or 2 metastatic sites (HR = 3.77 [95% CI, 1.27–11.40]; *P *= 0.016).

## Discussion

In patients with mCRC, bevacizumab has become one of highly recommended agents for first (5 mg/kg/2-wk) and second line (10 mg/kg/2-wk) chemotherapy. There have been many attempts to identify predictive biomarkers to help select those patients that will benefit from targeted agents, such as the association between the *KRAS *mutation status and survival outcomes in patients with mCRC treated with cetuximab [21]. As for bevacizumab, however, there are no predictive biomarkers identified to be associated with either treatment response or survival in patients with mCRC.

Both VEGF and CA9 are the products of hypoxia-induced pathways. VEGF, a target of bevacizumab, is a critical component of tumor angiogenesis. CA9, a transmembrane protein, converts carbon dioxide to bicarbonate and hydrogen, and thus regulates the microenvironment pH, as well as influences other processes such as cell-cell adhesion, proliferation, and invasion of tumor cells [22]. In this study, we demonstrated that the degree of CA9 expression was associated with a survival benefit after treatment with bevacizumab in previously treated patients with mCRC; however, VEGF expression was not related to the outcome of treatment in these patients. The degree of CA9 expression was divided into high and low using the expression score, which was defined as the intensity multiplied by the percent of stained area; an expression score ≥ 80 was considered to be high. The disease control rates were statistically higher, and the median PFS and OS were statistically lengthened in patients with low CA9 expression scores (< 80). Furthermore, CA9 expression remained significantly associated with the PFS after adjustment for other risk factors in multivariable analysis. The OS from initiation of first-line treatment, however, was not statistically different based on the CA9 expression scores (Figure 3). Thus, CA9 expression could be a predictive marker for a survival benefit, with bevacizumab treatment, rather than a prognostic marker, in patients with mCRC. The mechanism explaining the association of CA9 expression with bevacizumab resistance is unclear. One possible explanation might be suggested by the findings of Selvakumaran et al: where the antitumor effects of bevacizumab were suggested to be dependent on the susceptibility of tumors to hypoxia-induced apoptosis [23]. CA9 accelerates CO_2 _removal from the intracellular milieu as well as facilitates HCO_3 _^- ^recycling; thus, it serves to protect the tumor cells from acidosis [24]. Therefore, CA9 might play a role in the metabolic accommodation during hypoxia and may induce resistance to hypoxia-induced apoptosis by bevacizumab, although this hypothesis requires confirmation.

However, in contrast to CA9, VEGF expression was not associated with the clinical outcome of bevacizumab-based therapy. VEGF is a target molecule of bevacizumab and high levels of VEGF expression have been shown to be associated with a poor prognosis in patients with colorectal cancer [25]. In our study, however, there was no clinical significance associated with VEGF expression after bevacizumab treatment. In agreement with the results of the current study, a prior study reported that tumor VEGF expression was not a predictor of responsiveness to bevacizumab therapy [26].

The correlation between clinical outcomes and CA9 expression have been reported with several other malignancies including cancer of the cervix [27], lung [28], breast [29] and head and neck [30].

However, the most frequently reported studies associating CA9 expression with tumor behavior have been in patients with clear cell type renal cell carcinoma (RCC) [31-33]. CA9 is strongly expressed by RCC and it is known to be associated with clinical outcomes. Bui et al reported low CA9 expression (defined as ≤ 85% of tumor cells) was an independent predictor of poor prognosis after nephrectomy [32] and Atkins et al reported CA9 expression was a predictor of responses to interleukin-2 therapy [31]. Leibovich et al reported CA9 was not an independent predictor of clinical outcomes in patients with RCC after adjusting for the nuclear grade and tumor necrosis [34]; however, but it seemed to be true that CA9 expression was still one of the factors associated with RCC prognosis. In patients with RCC, a higher expression of CA9 was associated with better survival outcomes.

In terms of prediction of a survival benefit from bevacizumab, it has been reported that low CA9 expression was associated with better survival outcomes in patients with malignant astrocytoma treated with bevacizumab plus irinotecan [35]. From a phase II trial of bevacizumab plus irinotecan in patients with malignant astrocytoma, a comparative analysis was performed with several angiogenic factors including CA9 from tumor specimens by Sathornsumette et al. This phase II trial adopted a high dose regimen of bevacizumab, 10 mg/kg/2-wk [36]. They reported that a higher VEGF expression was associated with radiological responses to bevacizumab, but not with overall survival, and that the CA9 expression was associated with overall survival but not with radiological responses to bevacizumab. To the best of our knowledge, current study firstly suggested the predictive role of CA9 for DCR and PFS in bevacizumab-treated patients.

This study has another advantage showing that lower dose of bevacizumab at 5 mg/kg/2-wk could also result in clinical responses in the second line setting and even in the third or later line. Angiogenesis with VEGF is essential during the early stages of tumor progression; VEGF is expressed throughout the entire tumor life cycle. However, as more time elapses other factors in addition to VEGF itself become important at later stages of progression [37]. Therefore, it can be inferred that bevacizumab is more likely to be effective during the earlier stages of cancer, and that higher doses of bevacizumab may be needed to suppress the already activated VEGF pathways during later stages. Thus, the dose for phase III clinical trials using second-line treatment has been determined to be 10 mg/kg/2-wk, based on several preclinical and clinical studies that showed a dose-dependent effect of bevacizumab [15,38,39]. From a phase III trial comparing FOLFOX plus bevacizumab with FOLFOX plus placebo, second-line treatment with bevacizumab was demonstrated to be effective: the median PFS and OS were 7.3 months and 12.9 months with a 22.7% response rate [15]. However, recent clinical data on bevacizumab as first line treatment for non-small cell lung cancer and colorectal cancer demonstrated that there was no significant difference in the response rate and survival between lower dose and higher dose treatment groups[40,41] Although a phase II trial of lower dose of bevacizumab (5 mg/kg/2-wk) plus 5-FU/LV as a 3^rd^-line treatment failed to show clinical benefits [42], the lower dose still remained to be explored in second-line setting. In this study, 13 patients were treated with second line bevacizumab at 5 mg/kg/2-wk; 4 patients (30.8%) had confirmed partial responses and the median PFS was 5.0 months, which was not inferior to previous study. Furthermore, among 18 patients treated with third line or later bevacizumab, at 5 mg/kg/2-wk, 2 confirmed responses were observed (2/18, 11.1%) and the median PFS was lengthened to 4.5 months (95% CI, 1.0 – 8.3) for 11 patients that had a low CA9 expression score.

This study includes several limitations as follow: 1) the line of treatment with bevacizumab and concomitant chemotherapy were not constant, 2) this was a retrospective study with a small sample size, and the analysis had low statistical power, 3) patients without analyzable paraffin blocks were excluded, eliminating some of the patients treated with bevacizumab as second or later line therapy. Further evaluation of CA9 in patients enrolled in randomized prospective trials may confirm the use of CA9 as a marker in patients receiving bevacizumab therapy.

## Conclusion

In conclusion, CA9 IHC stain can be easily performed in clinical practice and it can be used to predict survival benefit in patients with previously treated mCRC who are considered to be treated with lower dose of bevacizumab as second or later lines. Further prospective, comparative analysis between the degree of CA9 expression and clinical outcomes in mCRC patients with 1^st ^line or higher dose of bevacizumab will be warranted.

## Competing interests

The authors declare that they have no competing interests.

## Authors' contributions

YSH is responsible for the study design. YSH and HJC collected the clinical data and drafted the manuscript. YSH, SYK and HJC revised the manuscript. HJC carried out tissue preparation and the pathology analysis; YSH, SYK, and KHJ were responsible for treatment and evaluation of the patients. JWP, JHO, HSC, BCK, and DKS participated in the collection of tissue samples and pathology data. DYK and HJC provided oversight of the analysis of data and drafting of the manuscript. All authors read and approved the final manuscript.

## Pre-publication history

The pre-publication history for this paper can be accessed here:

http://www.biomedcentral.com/1471-2407/9/246/prepub
